# Characterization of Particle-Size-Based Homogeneity and Mycotoxin Distribution Using Laser Diffraction Particle Size Analysis

**DOI:** 10.3390/toxins15070450

**Published:** 2023-07-06

**Authors:** Kai Zhang, Ivy Tran, Steven Tan

**Affiliations:** Office of Regulatory Science, Center for Food Safety and Applied Nutrition, Food and Drug Administration, 5001 Campus Drive, College Park, MD 20740, USA; ivy.tran@fda.hhs.gov (I.T.); steven.tan@fda.hhs.gov (S.T.)

**Keywords:** homogeneity, laser diffraction particle size analysis, mycotoxins

## Abstract

Sample homogeneity dictates whether analyzing a test portion of an entire sample can provide representative information about incurred mycotoxins. In this study, we evaluated particle-size-distribution-based homogeneity of laboratory mycotoxin samples using laser diffraction particle size analysis and International Organization for Standardization (ISO) Guide 35: 2017. Incurred whole corn, compound feed, peanut butter, and wheat flour (500 g each) were comminuted using wet, cryogenic, or dry milling. We used a sample dividing (riffling) device to obtain representative subsamples (25 g each) and developed a laser diffraction particle size analysis procedure by optimizing key parameters such as the refractive index, absorption, and stirring rate. The homogeneity of the particle size distribution within laboratory subsamples was characterized using the optimized laser diffraction procedure. An assessment of homogeneity was also performed for individual mycotoxins in each incurred matrix sample following the procedure described in ISO Guide 35. The concentrations of the incurred mycotoxins were determined using liquid chromatography–mass spectrometry (LC-MS). Within- and between-subsample variances of incurred aflatoxin B1 in peanut butter; deoxynivalenol in corn, compound feed, and wheat flour; and fumonisins in compound feed corroborated that when the particle size measurements were less than 850 µm, mycotoxins concentrations were consistent across independent test portions, which was confirmed using an analysis of variance (F-test). This study highlights the benefits of laser diffraction particle size analysis and suggests its use as a test procedure to evaluate homogeneity in new sample commodities.

## 1. Introduction

The determination of mycotoxins in field samples relies on sampling, sample preparation (comminution and homogenization), subsampling, and instrumental analysis (extraction, clean-up, identification, and quantitation). After a sample is collected, comminution by cutting, milling, grinding, and blending is typically conducted to minimize the impact of heterogenous distribution, i.e., “hot spots”, of mycotoxins [1]. For large sample sizes (10–20 kg), it is both cumbersome and impractical to analyze the entire sample for routine testing [2,3]. Therefore, a homogeneity assessment should be carefully planned and performed, ensuring that the subsampling and analysis of test portions reflect accurate and representative information on incurred mycotoxins, as the test portion usually accounts for a relatively small portion of the entire sample.

Sample homogeneity is a challenging and underexplored issue for mycotoxin analysis, especially for LC-MS-based multi-mycotoxin analysis. With advances in LC-MS technology, especially in terms of sensitivity, conventional extraction and concentration steps have been gradually replaced by dilution [4,5,6,7]. Smaller test portions offer operational and economic benefits, such as easy sample handling, savings in solvent consumption, and efficient turnaround time. Yet, these benefits come with a risk of compromising a representative and accurate determination of mycotoxins if sufficient homogeneity is not achieved.

A variety of protocols have been established for performing homogeneity assessments to determine whether a predefined subsampling plan and test portion would be adequate to provide representative information on target analytes. Both ISO Guide 35: 2017 [8] and the International Union of Pure and Applied Chemistry/International Organization for Standardization/Association of Official Agricultural Chemists International (IUPAC/ISO/AOAC) harmonized protocol provide specific guidance on the development of homogeneity and the associated statistical analysis of variance (ANOVA) [9,10]. The ISO Guide 35 has been used for the development of reference materials but is rarely adopted for routine sample analysis because of its lengthy and costly procedure. The IUPAC/ISO/AOAC similarly requires the replicate analysis of multiple samples but incorporates the use of the Horwitz ratio as a general model of precision [11]. Neither approach, however, can completely isolate variance associated with homogeneity from variance with sampling and/or the method of analysis [12].

Sieving has been the conventional and standard sizing measurement technique for corn samples containing mycotoxins [13]. Compared to other sizing techniques, sieving is affordable and suitable for a homogeneity assessment of samples with relatively large particle sizes, though it is difficult to apply to small, powdery particles with low flowability, such as flour, or cohesive and agglomerated food matrices, such as dried fruits or peanut butter. Manual sieving is simple but time-consuming and laborious, with poor reproducibility and low resolution [14].

Laser diffraction particle size analysis is an alternative methodology for size-dependent homogeneity assessments. With well-established mathematical theories [15,16,17] and commercially available software and hardware, the technique has been widely used in mining, food processing, pollution control, paint manufacturing, and in the pharmaceutical industry [18]. In sample preparation, particle size distributions may be used to characterize homogeneity [19,20]. For example, AOAC recommends a particle size threshold of 850 µm for routine sample preparation for mycotoxins, specifically aflatoxin [21]. For routine analysis, particle size measurements provide an alternative platform that, once optimized, can achieve the fast and reliable characterization of sample homogeneity within a few minutes [22,23].

Recognizing the important intermediary role of homogeneity assessment between sample preparation and instrumental analysis, we focused on routine mycotoxin sample preparation that addressed the challenge of obtaining small but representative test portions essential for LC-MS analysis. Encouraged by the promising but limited applications of laser diffraction particle size analysis in dried fruits [23,24], we considered the technique could be applied to more commonly consumed and representative food and feed matrices that are prone to mycotoxin contamination. However, the key parameters associated with laser diffraction particle size analysis such as dispersant, stirring rate, refractive index and absorption index should be thoroughly evaluated in representative matrices prepared via commonly used sample preparation procedures (e.g., dry, slurry and cryogenic milling). Furthermore, it would be worthwhile to confirm this new approach with an existing protocol, providing more confidence for the application of laser diffraction particle size analysis as a much-needed tool for routine sample analysis. This study aimed to address the following objectives:

- Develop a sizing measurement procedure for representative mycotoxin matrices using laser diffraction spectroscopy.
- Determine whether laser diffraction particle size analysis could provide results of the particle size distribution for homogeneity assessment in a practical and time-efficient manner.
- Confirm that particle size-based homogeneity assessment is consistent with ISO Guide 35.

## 2. Results and Discussion

### 2.1. Effects of Dispersion Parameters: Dispersant and Stirring Rate

**Figure 1:**
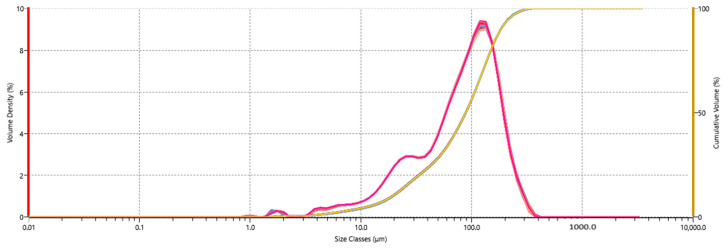
Overlay of 60 measurements of particle size distribution of wheat flour. Gaussian distribution: individual particle size class and corresponding volume density (left Y axis, red). Sigmoidal curve: cumulative particle size distribution and corresponding cumulative volume percentage (right Y axis, yellow).

In this study, samples were introduced into a measurement cell via a wet dispersion unit equipped with a variable speed stirrer. To achieve reliable particle size distribution results, one needs to consider key dispersion parameters such as dispersant and forces used to disperse agglomerated particles in a liquid suspension. Methanol was selected as the dispersant to wet the particle surfaces and separate touching particles [24]. Figure 1 is the overlay of particle size distribution data for 60 sequential measurements of wheat flour as the test matrix. Particle size distribution curves (gaussian) specify the corresponding volume density (left Y axis) of each individual particle size class, and cumulative undersize curves (sigmoidal) specify the corresponding Dv10, Dv50, and Dv90 values (right Y axis). The average Dv10, Dv50, and Dv90 were 19, 88, and 183 µm with RSDs < 1% (n = 60), suggesting the particle measurements were consistent in methanol. The same experiment was repeated using corn, compound feed, and peanut butter. No significant variability in particle size distribution was observed after the sample particles dispersed in methanol for up to 60 measurements. The corresponding Dv10, Dv50, and Dv90 values and RSDs (n = 60) were 15 (9), 153 (3), and 516 (3) in corn; 26 (3), 235 (2), 642 (3) in compound feed; and 4 (1), 19 (1), and 66 (4) in peanut butter, respectively.

**Figure 2:**
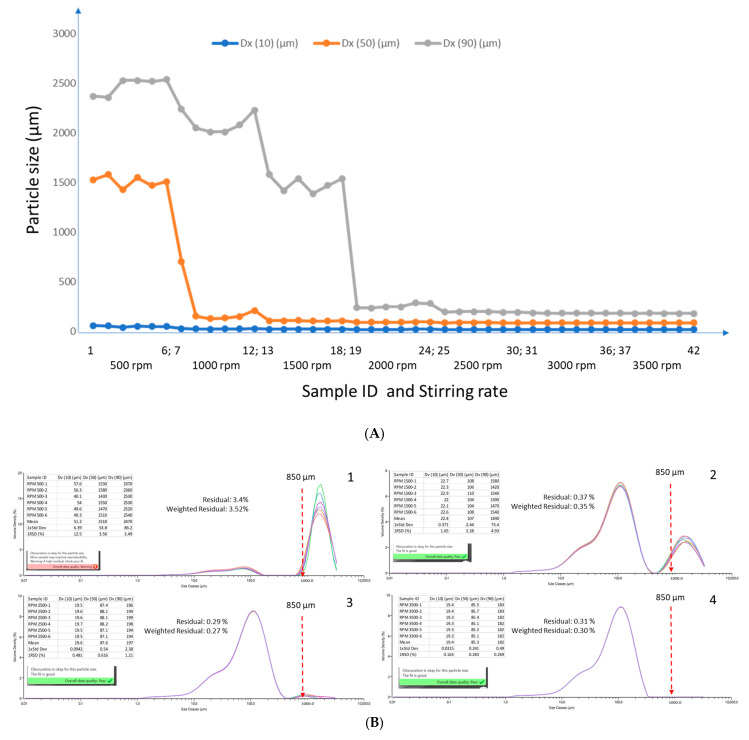
(**A**). Effect of stirring rate on particle size distribution of wheat flour. Samples 1–6 were measured at 500 rpm, 7–12 at 1000 rpm, 13–18 at 1500 rpm, 19–24 at 2000 rpm, 25–30 at 2500 rpm, 31–36 at 3000 rpm, and 37–42 at 3500 rpm. (**B**) (1–4). Repretative particle size distribution at 500 (1), 1500 (2), 2500 (3) and 3500 (4) rpm in a wheat flour sample.

Stirring is a common practice for dispersing agglomerated particles and creating a uniform suspension of particles in the dispersant. Without sufficient stirring, agglomeration, precipitation, and/or heterogenous suspension of particles could seriously influence light scattering patterns, leading to erroneous sizing data. Therefore, we compared the effect of stirring rate, ranging from 500 to 3500 rpm. Figure 2A illustrates that the particle size and distribution in the wheat flour sample decreased as a function of stirring rate up to 3500. This was highlighted by a clear trend showing that the overall particle size distribution (Dv10, Dv50, and Dv90) shifted toward much smaller ranges with increases in stirring rate. Figure 2B provides more details regarding the size distribution data, fitting, and data quality at 500, 1500, 2500 and 3500 rpm. At the lowest stirring rate, 500 rpm (Figure 2B, panel 1), due to agglomeration and heterogenous distribution, the residual and weighted residual values reached >3%, above the recommended threshold of 1–2% [25,26], suggesting unacceptable data quality and unrealistically large particle sizes (e.g., Dv90 > 2500 µm) that were poorly aligned with the light scattering patterns and models. At 1500, 2500, and 3500 rpm (Figure 2B, panels 2–4), the distribution curves became smooth and continuous, with the gradual disappearance of the dominant artifact peak (most frequently occurring size > 1000 µm) observed at 500 rpm. The fitting values decreased to approximately 0.3%, indicating satisfactory data quality. The most frequently occurring size centered around 100 µm due to a decrease in agglomerated particles and an increase in fines. As a result, we were confident that the selected dispersant, methanol, and stirring rate (3500 rpm) were suitable for the particle size analysis of food matrices used in this study.

### 2.2. Effects of Optical Parameters: Refractive Index, Absorption Index, and Obscuration

To use laser diffraction technology for particle size analysis, determination of the refractive index, a complex number consisting of a real number and an imaginary number, is required. The real number is the actual refractive index of the sample; the imaginary number is defined as the absorption, which is the amount of light absorbed by the sample. An imaginary value of 0 is often assigned to a transparent sample and a value of 1.0 or greater suggests that the sample is very opaque. Generally, actual refractive index and absorption index are not available for food matrices, so empirical refractive values (1.4, 1.6, and 1.8) and absorption values (0, 0.001, 0.01, 0.1, and 1) were selected for comparison using wheat flour as the representative food matrix.

The fitness of the data (residual and weighted residual) and relative standard deviation in particle size distribution was used to evaluate whether the selected refractive index, 1.6, and absorption, 0.01, should be optimized. Using the Dv10, Dv50, and Dv90 generated at 1.6 and 0.01 as the benchmark, Lin’s concordance correlation coefficient (Lin’s CCC) [27,28] was calculated to assess the impact of refractive index and absorption values on particle size distribution. A Lin’s CCC of >0.99 suggests the change in either or both optical properties has minimal impact, as two sets of Dv10, Dv50, and Dv90 have a strong concordance. Table 1 summarizes Dv10, Dv50, Dv90 and the corresponding fitness values generated at different refractive and absorption setting combinations. The changes in refractive index and/or absorption only resulted in minor variations in Dv10, Dv50, and Dv90 (<5% RSD). All Lin’s CCCs are >0.99 with residual values <1%, suggesting that experimental settings are robust, and that the data fit in the mathematical models coherently.

The effect of obscuration was evaluated at 10% and 20%—the low and the high ends of the normal operation range, 10–20%, recommended by the vendor. Obscuration is positively correlated with sample concentration in the measurement cell. When obscuration is set too low, with insufficient particles measured, the estimate of particle size distribution is likely to be exaggerated and variability would be high. When obscuration is set too high, with too many sample particles introduced into the measurement cell, particles are not well dispersed and distanced, leading to multi-scattering, which can change scattering patterns and skew particle size distribution results. At 20% obscuration, the only noticeable change was a 5% decrease (193 µm to 183 µm) in Dv90, while Dv10, Dv50, fitness, and Lin’s CCC remained consistent with those values at 10% obscuration.

### 2.3. Particle Size Distribution of Corn, Compound Feed, Peanut Butter, and Wheat Flour

The particle size distributions (Dv10, Dv50, and Dv90) of the ten subsamples of corn, compound feed, peanut butter, and wheat flour are summarized in Table 2. Overall, the Dv90 values were below 850 µm, recommended by AOAC [21], suggesting sufficient homogeneity in those samples. The Dv90 ranges of peanut butter (69–85 µm) and wheat flour (183–209 µm) were much smaller than that of corn (522–613 µm) or compound feed (522–674 µm). It is well known that the texture and taste of peanut butter are highly affected by particle size. Commercial peanut butter products are often made via multiple steps of grinding, allowing the final paste (≥98% by weight) to pass through a No. 200 sieve (74 µm) [29,30]. The quality of wheat flour is often evaluated based on its granularity (particle size). According to Codex, after milling, wheat flour (≥98% by weight) should pass through a No. 70 sieve (212 µm) [31]. Based on this information, it was not surprising to see very fine particles in these two matrices. In terms of particle size, the peanut butter and wheat flour samples used for the study would be considered homogenous and could be analyzed without extensive milling or grinding. However, prior to subsampling, mixing was still necessary, as wheat flour could absorb moisture from the air, forming clumps.

Whole corn kernels or compound feed had to be comminuted, as the initial sizes of kernels were much larger than 850 µm. The feed contains fibers, protein, and fat, making it difficult to form a homogenous fraction; therefore, it was prepared using cryogenic milling. This milling method achieved particle sizes with a Dv90 ranging between 522 and 613 µm. A few measurements had relatively large variability. For example, the RSDs of Dv90 in compound feed 3–2, peanut butter 1–1, 5–2, and 6–2 are >10%. Since all data points were retained without outlier analysis, sporadic large particles likely contributed to larger deviations in the measured Dv90 values. The observed within- and between-sample variability was probably also caused by the way in which test portions were taken. The sample divider cannot accurately collect 1 g aliquots, so each test portion was manually collected from the subsample and introduced into the dispersion unit. Increasing sample concentration in the measurement cell could also decrease variability at a higher probability of multi-scattering.

**Figure 3:**
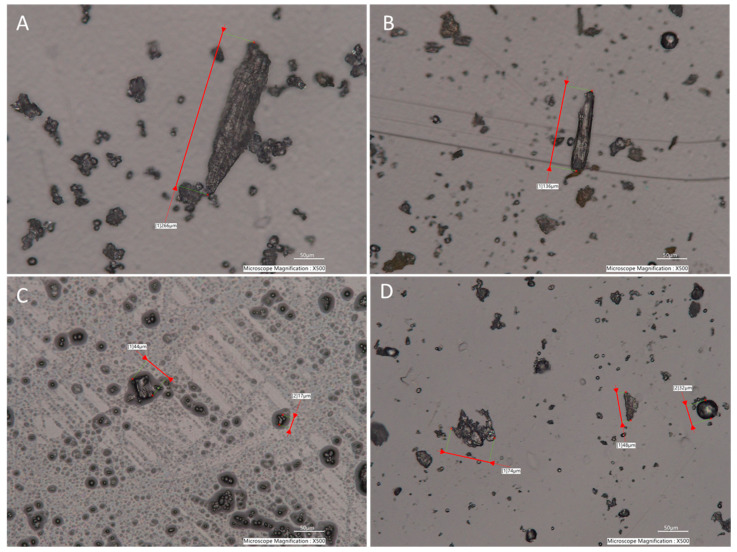
Microscope pictures of homogenized corn (**A**), compound feed (**B**), peanut butter (**C**), and wheat flour (**D**). (Photos were taken using a digital microscope, Keyence VHX-7000 Series.)

Particle shape and surface area also could affect scattering patterns, leading to inconsistent size distributions [32,33]. Microscopy photos (Figure 3) of the particles of corn, compound feed, peanut butter, and wheat flour highlight that the majority of the particles show non-spherical, irregular shapes, though size-wise, they are <850 µm. Therefore, it is important in future studies to evaluate approaches for sample milling and incorporate real-time image analysis to further understand the effect of particle shape on sizing measurement [34,35,36].

### 2.4. Confirmation of Homogeneity Using ISO Guide 35

ISO Guide 35 [8] provides specific guidance on the assessment of homogeneity in reference materials. Although its applicability to routine analysis is limited, the methodology provides a compound-selective approach to assess homogeneity in mycotoxin incurred samples. The particle size distribution of a comminuted sample could be used to assess the homogeneity of the sample, but one still needs to confirm whether the within- and between-sample variance of individual co-occurrent mycotoxins in the samples are small enough to ensure consistent quantitative analysis.

Following ISO Guide 35, we collected 30 test portions from ten subsamples of whole corn, compound feed, peanut butter, and wheat flour. Incurred deoxynivalenol in the test portions of whole corn, compound feed, and wheat flour; fumonisins and zearalenone in the test portions of compound feed; and aflatoxin B1 in the test portions of peanut butter were extracted and quantitated using automated sample preparation and LC-MS, followed by ANOVA to determine whether the homogeneous distribution of individual mycotoxins was achieved. If the calculated F value was less than the corresponding critical value (Fcrit) at a *p* > 0.05, the sample was considered homogeneous, as all the concentrations of the mycotoxin in tested subsamples were not statistically significant with the same mean. In other words, statistical analysis may be used to determine whether the sample should be further homogenized, or the results are representative for the whole sample.

Table 3, Table 4, Table 5, Table 6, Table 7, Table 8, Table 9 and Table 10 summarize descriptive statistics (sum (ppb), average (ppb), variance, standard deviation (SD), relative standard deviation (RSD %)) and ANOVA of incurred mycotoxins. With the exception of fumonisin B2 in a compound feed sample, aflatoxin B1 (peanut butter), deoxynivalenol (corn, feed, and wheat flour), fumonisin B1 (feed), fumonisin B3 (feed), and zearalenone (feed) were considered homogenously distributed in the comminuted samples of the various test matrices, as the calculated F values were smaller than the critical F value with a value *p* > 0.05, suggesting no significant differences among the test samples. These results aligned consistently with the particle-size-based homogeneity assessment.

Interestingly, fumonisin B2 failed the F test. Although variability in homogeneity, sampling, test portion size, and analytical method could contribute to the overall uncertainty in the concentration of fumonisin B2, it is unlikely that the distribution of fumonisin B2 would be significantly different from that of fumonisin B1 or B3 in the same feed product. We speculated that co-eluted interferences from the feed matrix caused the large variability due to the limited cleanup used for sample preparation [37]. This leads to a possible explanation, as the statistical test of ISO Guide 35 cannot separate variance associated with homogeneity from that with analytical measurements. Fumonisin B2 was additionally evaluated using the IUPAC/ISO/AOAC harmonized protocol. Using any two sets of data, the corresponding sampling variance was less than the critical value (Table 11), suggesting the distribution of fumonisin B2 in the sample is considered to have “sufficient homogeneity” [8,9,10].

In naturally contaminated agricultural commodities [38,39,40], heterogeneous distribution may be influenced by mycotoxin type (e.g., aflatoxins vs. deoxynivalenol or fumonisins). In finished food and feed products, sources of the starting materials and processing make the distribution even more complicated and unpredictable [41], requiring different sample preparation strategies to achieve sufficient homogeneity. According to Gy’s sampling theory, fundamental sampling error is positively correlated to the cube of sample particle size but is inversely proportional to the sampling mass (size of test portion). In the context of this work, at particle size thresholds (<850 µm), homogeneity was determined to be independent of mycotoxin and matrix type. However, additional investigations are warranted to support the acceptance of a single particle size threshold to estimate the homogeneity (distribution) of individual mycotoxins in different matrix types.

## 3. Conclusions

Due to the heterogenous distribution of mycotoxins in foods, samples to be analyzed for mycotoxins are required to be comminuted prior to extraction. The required homogeneity threshold of samples is often defined by the sample particle sizes (e.g., sample particles passed through a #20 sieve, 850 µm). In this work, the sample homogeneity in small-scale laboratory samples was characterized using an optimized laser diffraction particle size method and compared to ISO Guide 35: 2017. The results demonstrated that matrices with Dv90 < 850 um could be considered homogenous with respect to the analyzed aflatoxins, deoxynivalenol, and fumonisins. Although particle size analysis can only be used to estimate the distribution of individual mycotoxins based on the assumption that all incurred mycotoxins share similar distribution patterns after comminution, the methodology provides an efficient test procedure to characterize new sample commodities and confirm consistency in sample fractionation for routine analysis.

## 4. Materials and Methods

### 4.1. Sample Comminution and Subsampling

Whole corn, commercial compound feed, peanut butter, and wheat flour (white) (500 g/each) were collected from local stores and used for this study. The whole corn and compound feed samples were stored at −80 °C overnight. On the next day, the two samples were mixed with dry ice and comminuted using a Retsch MM 500 mixer mill (Verder Scientific Inc., Newtown, PA, USA) equipped with two grinding chambers until a powdery consistency was achieved. The grinding time and speed were set at 5 min and 35 Hz, respectively. Comminuted samples were transferred into polypropylene bags and stored at −20 °C in dark conditions. After dry ice was sublimed, the samples were used for subsampling. The wheat flour and the peanut butter were mixed individually using a Blixer 4 blender for 3 min (Robot Coupe, Inc., Ridgeland, MS, USA).

**Figure 4:**
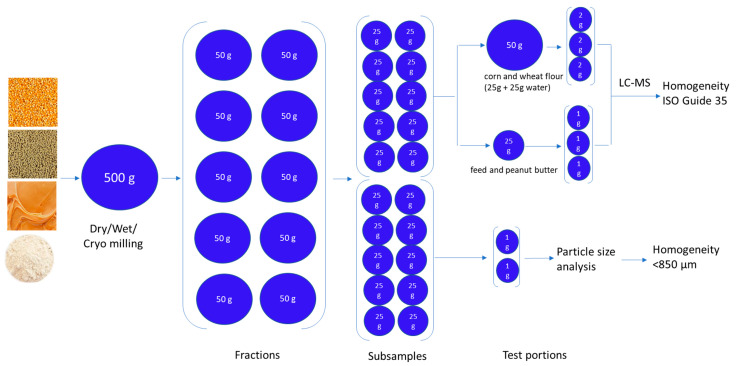
Hierarchical flowchart of sample preparation: fractions, subsamples, and test portions.

After comminution, fractions, subsamples, and test portions (analytical samples) were collected via a three-step process. Step 1: Divide the 500 g sample into ten fractions of 50 g. Step 2: Divide each 50 g fraction into two subsamples of 25 g. Step 3: Collect three test portions from each of the ten 25 g subsamples for LC-MS analysis and collect two test portions from the other ten 25 g subsamples for particle size analysis. The hierarchical design of sample preparation, including the collection of fractions, subsamples, and test portions is illustrated in Figure 4 and described as follows.

Step 1. The comminuted corn, compound feed and wheat flour samples were mixed to break clumps and divided into ten fractions using a Retsch PT-100 sample divider (Verder Scientific, Newtown, PA, USA) and IKA 100 mL grinding chambers (IKA Inc., Wilmington, NC, USA). The riffling device (sample divider) included a vibratory hopper, a feeder, and a carousel with eight collection containers. For each batch, 100 g of the sample was divided into eight aliquots. The vibration rate and rotational parameters were optimized to ensure that the relative standard deviation (RSD) of the masses of the eight aliquots (12.5 ± 0.5 g/each) was less than 5%. After the 500 g sample was divided into 40 aliquots, 4 aliquots were randomly combined and mixed in an IKA 100 mL grinding chamber. In this way, ten 50 g sample fractions (50 ± 2 g/each) were obtained.

Step 2. Each 50 g fraction was divided into two subsamples (25 ± 1 g each), saved in IKA grinding chambers. This way, there were twenty 25 g subsamples for each matrix.

Step 3. For corn and wheat flour, the following procedures were used to collect test portions for LC-MS analysis and particle size analysis, respectively:

LC-MS test portions: We selected ten 25 g subsamples and mixed each subsample with 25 g of water following a slurry procedure [42,43]. The subsample and water were blended using an IKA tube mill. Once the slurry (thick paste) was formed, test portions were weighed out (2 g each containing 1 g dry sample + 1 g water) using a laboratory scoop and a balance from the resulting slurry into 15 mL disposable screw-capped polypropylene centrifuge tubes. Three test portions were collected from each prepared slurry sample and analyzed using LC-MS [43,44]. In total, 30 test portions were collected from the 10 subsamples following the ISO-Guide 35.

Particle size analysis test portion: The other ten 25 g subsamples were used for particle size analysis. Two test portions (1 g each) were collected from each 25 g subsample, and each test portion was measured with a particle size analyzer. Before we took the two test portions from a 25 g subsample, we slowly rotated and turned the sample tube multiple times. The first test portion was taken using a scoop soon after mixing to ensure no settling had occurred. We repeated the same steps to collect the next test portion.

As it was difficult to make slurries for the compound feed, after Step 2, we selected ten 25 g subsamples and collected three test portions (1 g each) from each of the subsamples for LC-MS analysis. Prior to test portion collection, we slowly rotated and turned the sample tube multiple times. The first test portion was taken using a scoop soon after mixing to ensure no settling had occurred. We repeated the same steps to collect the next two test portions. The test portions for particle size analysis were collected from the other ten 25 g subsamples as described above. The only difference is we only collected two test portions (1 g each) from each of the subsamples for particle size analysis.

The peanut butter could not be divided by the riffling device, so its ten fractions (50 g each) were prepared manually. After the first fraction was collected from the Blixer 4 blender, the remaining peanut butter was mixed using a laboratory spatula followed by the collection of the second fraction. We repeated the same procedure until the ten fractions were collected. The ten 50 g fractions were further divided into twenty 25 g subsamples. Ten 25 g subsamples were used for LC-MS analysis and three test portions (1 g each) were directly collected from each subsample without using the slurry procedure. The other ten 25 g subsamples were used for particle size analysis. Two test portions (1 g each) were collected from each subsample.

The above test portions collected for LC-MS analysis were prepared as described in a previous study and an FDA compendial method [42,43]. In brief, 13C uniformly labelled mycotoxins (13C-aflatoxin B1, B2, G1, and G2; 13C-fumonisin B1, B2, and B3; 13C-deoxynivalenol; 13C-HT-2/T-2; 13C-zearalenone; and 13C-ochratoxin A) were spiked into each test portion, followed by extraction using 50% acetonitrile, centrifugation, and filtration.

The test portions collected above for particle size analysis were pre-wetted using the dispersant (methanol). Transfer pipettes were used to add methanol to the samples, which were blended and pipetted up and down to ensure complete wetting. Care had to be taken to measure the sample as soon as possible after the slurry was created to eliminate any excess dissolution.

### 4.2. Particle Size Analysis

Sample particle size measurements were conducted using a Mastersizer 3000 (Malvern Panalytical, Malvern, UK). Samples were dispersed and introduced into the Mastersizer 3000 using a Hydro LV filled with ~500 mL methanol as the dispersant. The stirrer speed was set at 3500 rpm. The particle refractive index and particle absorption index were set at 1.6 and 0.01, respectively. The refractive index of methanol was 1.32. The particle size was calculated on a volume basis using Malvern Mastersizer software (version 3.81). The analysis settings used were the General Purpose model with the Irregular Shape Mode. Background light scattering signals from the system and the dispersant (methanol) were measured prior to the addition of the sample. For the collected corn, compound feed, peanut butter and wheat flour test portions, we used transfer pipettes to add the dispersant (methanol) to pre-wet the sample and mixed the test portion into a thick slurry. We pipetted up and down to ensure complete wetting. Care had to be taken to measure the sample as soon as possible after the slurry was created to eliminate any excess dissolution. Next, individual test portions were added to the dispersion unit. We initiated the particle size measurement procedure when the obscuration value was within the manufacturer’s recommended range (10–20%). Six repeated measurements were made on each sample. Data were collected at a rate of 10 kHz. CRM3100 (Malvern Panalytical, Malvern, UK) was analyzed as the QC sample to demonstrate instrument performance. To ensure sample particles were stable in methanol without significant morphological changes during analysis, we performed a repeatability test, measuring replicate test portions of the same wheat flour sample.

### 4.3. Confirmation of Homogeneity Using ISO GUIDE 35

To confirm sample homogeneity characterized using particle size distribution, one still needs to confirm whether one sizing threshold would be good for all analytes in different food matrices. The co-occurrence of mycotoxins in a sample matrix has been well documented; however, the question of whether homogeneity should be evaluated for each mycotoxin–matrix pair remains unanswered. To address this question, we acquired compound-specific distribution data following ISO Guide 35.

### 4.4. LC-MS Analysis

To determine the homogeneity of individual mycotoxin samples, LC-MS was used to analyze test portions collected from each subsample following a previously validated LC-MS procedure [26]. Prior to LC-MS analysis, the test portions were prepared using a Chemspeed XL Swing automated sample preparation system [44].

### 4.5. Statistical Analysis

Microsoft Excel was used to perform a one-way ANOVA of detected mycotoxins in whole corn, compound feed, peanut butter, and wheat flour. Homogeneity in the 10 subsamples of each matrix was evaluated based on ANOVA results. At a *p* value of >0.05, if the calculated F ratio (Fcal) was less than the corresponding critical value (Fcrit), the sample was defined as homogeneous [8].

## Figures and Tables

**Table 1:** Cumulative particle size distribution at Dv10, Dv50, and Dv90 (% relative standard deviation, n = 6) in wheat flour at different obscuration, refractive index, and absorption index settings.

Obscuration	Refractive Index	Absorption Index	Dv10 (µm)	Dv50 (µm)	Dv90 (µm)	Lin’s CCC	Residual	Weighted Residual
10	1.4	0.01	18 (5)	79 (3)	192 (2)	0.9980	0.30%	0.36%
10	1.6	0	20 (5)	80 (3)	193 (2)	0.9981	0.31%	0.31%
10	1.6	0.001	21 (5)	80 (3)	193 (2)	0.9981	0.29%	0.29%
10	1.6	0.01	19 (5)	80 (3)	193 (2)	1.0000	0.33%	0.32%
10	1.6	0.1	19 (5)	79 (3)	193 (2)	0.9980	0.27%	0.30%
10	1.6	1	18 (5)	79 (3)	192 (2)	0.9979	0.26%	0.26%
10	1.8	0.01	19 (5)	79 (3)	193 (2)	0.9999	0.30%	0.28%
20	1.4	0.01	18 (1)	85 (1)	182 (1)	0.9943	0.32%	0.38%
20	1.6	0.01	21 (1)	87 (1)	183 (1)	0.9943	0.33%	0.34%
20	1.8	0.01	20 (0.5)	86 (0.5)	183 (1)	0.9943	0.34%	0.33%

**Table 2:** Cumulative particle size distribution at Dv10, Dv50, and Dv90 (% relative standard deviation, n = 6) in corn, compound feed, peanut butter, and wheat flour.

Sample #- Test Portion #	Corn			Compound Feed			Peanut Butter			Wheat Flour		
	Dv10 (µm)	Dv50 (µm)	Dv90 (µm)	Dv10 (µm)	Dv50 (µm)	Dv90 (µm)	Dv10 (µm)	Dv50 (µm)	Dv90 (µm)	Dv10 (µm)	Dv50 (µm)	Dv90 (µm)
1–1	14 (2)	161 (4)	577 (2)	27 (3)	244 (2)	665 (2)	4 (1)	17 (1)	85 (12)	19 (5)	80 (3)	193 (2)
1–2	14 (2)	161 (3)	564 (2)	27 (3)	244 (3)	670 (4)	4 (1)	17 (1)	79 (8)	17 (2)	73 (2)	186 (2)
2–1	14 (3)	165 (5)	582 (3)	25 (5)	219 (7)	614 (5)	4 (1)	17 (1)	76 (6)	17 (7)	89 (6)	209 (2)
2–2	13 (1)	147 (2)	557 (2)	24 (5)	202 (4)	552 (6)	4 (1)	17 (1)	76 (9)	15 (2)	79 (2)	199 (1)
3–1	14 (3)	160 (6)	561 (3)	25 (2)	208 (2)	566 (4)	4 (1)	17 (1)	73 (6)	17 (6)	85 (6)	203 (3)
3–2	13 (1)	150 (3)	561 (3)	24 (5)	209 (5)	588 (12)	3 (1)	17 (1)	75 (5)	15 (3)	75 (3)	190 (2)
4–1	14 (2)	163 (4)	577 (2)	28 (5)	239 (4)	627 (5)	4 (1)	17 (1)	73 (8)	16 (6)	79 (6)	198 (3)
4–2	13 (3)	145 (4)	554 (2)	29 (4)	242 (2)	625 (4)	4 (1)	16 (1)	72 (6)	15 (2)	74 (3)	187 (2)
5–1	14 (3)	172 (8)	575 (4)	24 (2)	234 (2)	619 (2)	4 (1)	17 (1)	73 (5)	17 (6)	86 (6)	205 (3)
5–2	13 (1)	153 (3)	549 (2)	25 (2)	239 (1)	630 (3)	4 (1)	17 (1)	74 (14)	15 (2)	75 (2)	189 (1)
6–1	14 (2)	164 (4)	591 (4)	27 (3)	232 (3)	671 (4)	4 (1)	17 (1)	73 (4)	17 (7)	86 (7)	205 (3)
6–2	14 (2)	154 (4)	589 (4)	24 (4)	215 (3)	617 (10)	4 (1)	17 (2)	80 (23)	15 (2)	74 (3)	191 (2)
7–1	14 (3)	159 (6)	568 (2)	24 (3)	218 (3)	658 (7)	4 (1)	17 (1)	72 (9)	27 (7)	81 (5)	183 (1)
7–2	13 (1)	145 (3)	563 (2)	24 (1)	216 (2)	629 (3)	4 (1)	17 (1)	72 (2)	21 (7)	75 (1)	185 (1)
8–1	14 (3)	160 (6)	613 (2)	27 (4)	233 (3)	634 (8)	4 (1)	16 (1)	71 (7)	17 (4)	85 (5)	201 (3)
8–2	14 (1)	154 (2)	594 (1)	27 (1)	235 (2)	622 (3)	4 (1)	16 (1)	69 (5)	15 (2)	75 (2)	186 (1)
9–1	14 (2)	162 (5)	531 (2)	27 (2)	227 (1)	577 (2)	4 (1)	16 (1)	70 (5)	17 (6)	85 (7)	204 (3)
9–2	14 (2)	149 (4)	522 (3)	27 (4)	236 (3)	609 (7)	4 (1)	16 (1)	70 (2)	15 (2)	74 (3)	188 (2)
10–1	14 (3)	179 (3)	596 (2)	27 (4)	224 (3)	587 (3)	4 (1)	17 (1)	72 (5)	16 (1)	83 (5)	203 (6)
10–2	14 (1)	171 (3)	599 (2)	26 (3)	219 (1)	565 (2)	4 (1)	16 (1)	71 (6)	15 (1)	74 (2)	188 (3)
Range (µm)	13-14	145-179	522-613	24-27	202-244	552-671	3-4	16-17	69-85	15-27	73-89	183-209
Grand mean (RSD %)	14 (3)	159 (6)	571 (4)	26 (6)	267 (6)	616 (6)	4 (6)	17 (3)	74 (5)	17 (17)	79 (7)	195 (4)
QC (CRM 3310)	39 (1)	74 (1)	111 (1)	41 (1)	75 (1)	110 (1)	39 (1)	73 (1)	111 (3)	41 (1)	76 (1)	117 (3)

Grand mean was calculated using the 20 individual measurements. Certified Dv10, Dv50, and Dv90 values (RSD) of CRM 3310: 39 (5), 73 (4), and 105 (6).

**Table 3:** ANOVA of deoxynivalenol in corn.

Subsample	Test Portion Analyzed (3)	Sum (ng/g)	Average	Variance	SD	RSD (%)
1	3	1026.99	342.33	200.89	14.17	4.14
2	3	967.31	322.47	19.36	4.40	1.36
3	3	923.40	307.80	923.23	30.38	9.87
4	3	1028.10	342.70	36.27	6.02	1.76
5	3	1020.81	340.27	271.36	16.47	4.84
6	3	1032.09	344.03	96.20	9.81	2.85
7	3	1017.39	339.13	707.56	26.60	7.84
8	3	1049.19	349.73	42.89	6.55	1.87
9	3	1004.91	334.97	175.57	13.25	3.96
10	3	980.91	326.97	10.57	3.25	0.99
ANOVA						
Source of Variation	SS	df	MS	F	*p*-value	F crit
Between Groups	4254.02	9	472.67	1.90	0.11	2.39
Within Groups	4967.85	20	248.39			
Total	9221.872	29				

**Table 4:** ANOVA of deoxynivalenol in compound feed.

Subsample	Test Portion Analyzed (3)	Sum (ng/g)	Average (ng/g)	Variance	SD	RSD (%)
1	3	2461.30	820.43	826.80	28.75	3.50
2	3	2382.00	794.00	1806.79	42.51	5.35
3	3	2501.80	833.93	498.49	22.33	2.68
4	3	2491.80	830.60	3175.69	56.35	6.78
5	3	2349.80	783.27	1432.90	37.85	4.83
6	3	2513.90	837.97	1361.65	36.90	4.40
7	3	2630.50	876.83	2264.90	47.59	5.43
8	3	2518.90	839.63	660.04	25.69	3.06
9	3	2481.80	827.27	2085.32	45.67	5.52
10	3	2503.10	834.37	5407.14	73.53	8.81
ANOVA						
Source of Variation	SS	df	MS	F	*p*-value	F crit
Between Groups	17,749.31	9	1972.15	1.01	0.46	2.39
Within Groups	39,039.49	20	1951.97			
Total	56,788.80	29				

**Table 5:** ANOVA of fumonisin B1 in compound feed.

Subsample	Test Portion Analyzed (3)	Sum (ng/g)	Average (ng/g)	Variance	SD	RSD (%)
1	3	789.10	263.03	1655.42	40.69	15.47
2	3	717.70	239.23	599.86	24.49	10.24
3	3	721.90	240.63	108.70	10.43	4.33
4	3	770.00	256.67	1102.94	33.21	12.94
5	3	658.40	219.47	121.96	11.04	5.03
6	3	794.30	264.77	283.42	16.84	6.36
7	3	838.30	279.43	20.84	4.57	1.63
8	3	781.80	260.60	604.11	24.58	9.43
9	3	804.10	268.03	59.62	7.72	2.88
10	3	689.50	229.83	172.76	13.14	5.72
ANOVA						
Source of Variation	SS	df	MS	F	*p*-value	F crit
Between Groups	9695.38	9	1077.26	2.28	0.06	2.39
Within Groups	9459.32	20	472.97			
Total	19,154.70	29				

**Table 6:** ANOVA of fumonisin B2 in compound feed.

Subsample	Test Portion Analyzed (3)	Sum (ng/g)	Average (ng/g)	Variance	SD	RSD (%)
1	3	185.71	61.90	89.87	9.48	15.31
2	3	176.85	58.95	56.47	7.51	12.75
3	3	181.03	60.34	3.74	1.93	3.20
4	3	194.75	64.92	124.56	11.16	17.19
5	3	151.60	50.53	4.72	2.17	4.30
6	3	194.37	64.79	46.49	6.82	10.52
7	3	200.90	66.97	4.24	2.06	3.07
8	3	187.33	62.44	15.98	4.00	6.40
9	3	214.29	71.43	28.88	5.37	7.52
10	3	170.79	56.93	0.43	0.66	1.16
ANOVA						
Source of Variation	SS	df	MS	F	*p*-value	F crit
Between Groups	897.79	9	99.75	2.66	0.03	2.39
Within Groups	750.73	20	37.54			
Total	1648.52	29				

**Table 7:** ANOVA of fumonisin B3 in compound feed.

Subsample	Test Portion Analyzed (3)	Sum (ng/g)	Average (ng/g)	Variance	SD	RSD (%)
1	3	76.12	25.37	10.23	3.20	12.61
2	3	61.48	20.49	0.89	0.94	4.61
3	3	69.74	23.25	0.89	0.94	4.05
4	3	72.27	24.09	11.26	3.36	13.93
5	3	55.11	18.37	0.85	0.92	5.01
6	3	64.86	21.62	9.79	3.13	14.47
7	3	67.88	22.63	2.84	1.69	7.45
8	3	71.81	23.94	1.34	1.16	4.84
9	3	68.95	22.98	9.84	3.14	13.65
10	3	63.23	21.08	11.92	3.45	16.38
ANOVA						
Source of Variation	SS	df	MS	F	*p*-value	F crit
Between Groups	112.20	9	12.47	2.08	0.08	2.39
Within Groups	119.68	20	5.98			
Total	231.88	29				

**Table 8:** ANOVA of zearalenone in compound feed.

Subsample	Test Portion Analyzed (n = 3)	Sum (ng/g)	Average (ng/g)	Variance	SD	RSD (%)
1	3	168.57	56.19	25.78	5.08	9.04
2	3	195.38	65.13	9.66	3.11	4.77
3	3	175.26	58.42	2.57	1.60	2.75
4	3	157.28	52.43	74.36	8.62	16.45
5	3	158.96	52.99	8.87	2.98	5.62
6	3	185.42	61.81	45.18	6.72	10.88
7	3	178.35	59.45	6.07	2.46	4.14
8	3	160.04	53.35	88.88	9.43	17.67
9	3	178.08	59.36	13.55	3.68	6.20
10	3	170.70	56.90	83.01	9.11	16.01
ANOVA						
Source of Variation	SS	df	MS	F	*p*-value	F crit
Between Groups	450.46	9	50.05	1.40	0.25	2.39
Within Groups	715.85	20	35.79			
Total	1166.31	29				

**Table 9:** ANOVA of aflatoxin B1 in peanut butter.

Subsample	Test Portion Analyzed (n = 3)	Sum (ng/g)	Average (ng/g)	Variance	SD	RSD (%)
1	3	2.74	0.91	0.004	0.06	6.88
2	3	3.04	1.01	0.002	0.05	4.88
3	3	3.02	1.01	0.022	0.15	14.76
4	3	2.58	0.86	0.018	0.14	15.78
5	3	2.51	0.84	0.036	0.19	22.78
6	3	2.96	0.99	0.009	0.10	9.70
7	3	2.65	0.88	0.027	0.17	18.74
8	3	2.80	0.93	0.002	0.04	4.26
9	3	2.45	0.82	0.006	0.08	9.73
10	3	2.70	0.90	0.004	0.06	7.11
ANOVA						
Source of Variation	SS	df	MS	F	*p*-value	F crit
Between Groups	0.13	9	0.01	1.10	0.41	2.39
Within Groups	0.26	20	0.01			
Total	0.39	29				

**Table 10:** ANOVA of deoxynivalenol in wheat flour.

Subsample	Test Portion Analyzed (n = 3)	Sum (ng/g)	Average (ng/g)	Variance	SD	RSD (%)
1	3	339.90	113.30	75.73	8.70	7.68
2	3	375.09	125.03	16.06	4.01	3.21
3	3	358.99	119.63	8.36	2.89	2.42
4	3	365.49	121.83	21.49	4.64	3.81
5	3	377.10	125.70	0.76	0.87	0.69
6	3	368.61	122.87	30.49	5.52	4.49
7	3	368.91	122.97	2.24	1.50	1.22
8	3	355.50	118.50	0.13	0.36	0.30
9	3	368.70	122.90	24.52	4.95	4.03
10	3	360.39	120.13	55.61	7.46	6.21
ANOVA						
Source of Variation	SS	df	MS	F	*p*-value	F crit
Between Groups	352.05	9	39.12	1.66	0.16	2.39
Within Groups	470.82	20	23.54			
Total	822.87	29				

**Table 11:** Concentrations (ng/g) of fumonisin B2 in the compound feed and homogeneity assessment ^a^.

Subsample #	Test Portion Set #		
	1	2	3
1	55.47	57.45	72.79
2	56.39	67.41	53.05
3	60.81	58.22	62.00
4	58.21	58.74	77.80
5	49.83	52.97	48.80
6	59.96	61.82	72.59
7	68.80	64.74	67.36
8	63.15	66.04	58.14
9	72.78	65.51	76.00
10	56.53	57.69	56.57
	Set 1 and 2	Set 1 and 3	Set 2 and 3
Analytical variance	0.000011	0.000045	0.000057
Between-sample variance	0.003352	0.006687	0.000141
Allowable between-sample variance	0.000020	0.000020	0.000020
Sampling variance	0.000022	0.000033	0.000007
Critical value	0.000048	0.000082	0.000094

^a^ Homogeneity assessment was performed following the protocol by Thompson et al., 2006 [10]. Sampling variance < critical value suggests sufficiently homogenous.

## Data Availability

Data available on request following Public Access to Results of FDA-Funded Scientific Research.

## References

[1] Campbell A.D., Whitaker T.B., Pohland A.E., Dickens J.W., Park D.L. (1986). Sampling, sample preparation, and sampling plans for foodstuffs for mycotoxin analysis. Pure Appl. Chem..

[2] European Commission Commission Regulation (EC) No 401/2006 of 23 February 2006 Laying Down the Methods of Sampling and Analysis for the Official Control of the Levels of Mycotoxins in Foodstuffs. https://eur-lex.europa.eu/legal-content/EN/TXT/PDF/?uri=CELEX:32006R0401&from=EN.

[3] Food and Drug Administration The Investigations Operations Manual (IOM) Chapter 4—Sampling. https://www.fda.gov/media/75243/download.

[4] Crosby N.T. (1984). Review of current and future analytical methods for the determination of mycotoxins?. Food Addit. Contam..

[5] Krska R., Schubert-Ullrich P., Molinelli A., Sulyok M., MacDonald S., Crews C. (2008). Mycotoxin analysis: An update. Food Addit. Contam. Part A.

[6] Berthiller F., Brera C., Crews C., Iha M.H., Krska R., Lattanzio V., Macdonald S., Malone R., Maragos C., Solfrizzo M. (2016). Developments in mycotoxin analysis: An update for 2015–2016. World Mycotoxin J..

[7] Zhang K., Banerjee K. (2020). A Review: Sample Preparation and Chromatographic Technologies for Detection of Aflatoxins in Foods. Toxins.

[8] (2017). Reference Materials—Guidance for Characterization and Assessment of Homogeneity and Stability.

[9] Thompson M., Wood R. (1993). The international harmonized protocol for the proficiency testing of (chemical) analytical laboratories (Technical Report). Pure Appl. Chem..

[10] Thompson M., Ellison S.L.R., Wood R. (2006). The international harmonized protocol for the proficiency testing of (chemical) analytical laboratories (Technical Report). Pure Appl. Chem..

[11] Albert R., Horwitz W. (1997). A Heuristic Derivation of the Horwitz Curve. Anal. Chem..

[12] Fearn T., Thompson M. (2001). A new test for ‘sufficient homogeneity’. Analyst.

[13] AOAC International Official Method 965.22, Sorting Corn Grits. http://www.aoacofficialmethod.org/index.php?main_page=product_info&cPath=1&products_id=1846.

[14] Allen T., Terence Allen T., Sampling P., Determination P.S. (2003). 4—Particle Size Analysis by Sieving.

[15] De Boer G.B.J., de Weerd C., Thoenes D., Goossens H.W.J. (1987). Laser Diffraction Spectrometry: Fraunhofer Diffraction Versus Mie Scattering. Part. Part. Syst. Charact..

[16] Lock J.A., Gouesbet G. (2009). Generalized Lorenz-Mie theory and applications. J. Quant. Spectrosc. Radiat. Transf..

[17] Wriedt T., Hergert W., Wriedt T. (2012). Mie Theory: A Review. The Mie Theory.

[18] Black D.L., McQuay M.Q., Bonin M.P. (1996). Laser-based techniques for particle-size measurement: A review of sizing methods and their industrial applications. Prog. Energy Combust. Sci..

[19] Pitard F.F. (1989). Pierre Gy’s Sampling Theory and Sampling Practice.

[20] Gerlach R.W., Nocerino J.M. (2003). EPA/600/R-03/027 Guidance for Obtaining Representative Laboratory Analytical Subsamples from Particulate Laboratory Samples.

[21] AOAC International Official Methods 977.16, Sampling for Aflatoxins—Preparation for Sample Procedure. http://www.aoacofficialmethod.org/index.php?main_page=product_info&products_id=2065.

[22] Ferraris C., Bullard J., Hackley V. (2006). Particle Size Distribution by LASER Diffraction Spectrometry: Application to Cementitious Powders. Aiche J..

[23] Zhang K., Tan S., Xu D. (2022). Determination of Mycotoxins in Dried Fruits Using LC-MS/MS—A Sample Homogeneity, Troubleshooting and Confirmation of Identity Study. Foods.

[24] Tan S., Zhang K., Martinez C.L. (2021). Laser Diffraction Particle Size Analysis of Mycotoxin Samples. Poster Presentation. The FDA Annual Student Scientific Research Day. https://www.fda.gov/science-research/fda-stem-outreach-education-and-engagement/laser-diffraction-particle-size-analysis-mycotoxin-samples.

[25] De Cleyn E., Holm R., Mooter G.V.D. (2019). Size Analysis of Small Particles in Wet Dispersions by Laser Diffractometry: A Guidance to Quality Data. J. Pharm. Sci..

[26] Malvern Panalytical (2013). Mastersizer-3000-User-Manual-English-MAN0474-2-1. https://www.malvernpanalytical.com/en/learn/knowledge-center/user-manuals/man0474en.

[27] Lin L.I.-K. (1989). A Concordance Correlation Coefficient to Evaluate Reproducibility. Biometrics.

[28] Lin L.I. (2000). A note on the concordance correlation coefficient. Biometrics.

[29] Hlavacek R.G., Freeman W.G. (1968). Low-heat, 200 mesh milling provides smooth, uniform product. Food Process. Mark..

[30] Mohd Rozalli N.H., Chin N.L., Yusof Y.A. (2015). Grinding characteristics of Asian originated peanuts (*Arachishypogaea* L.) and specific energy consumption during ultra-high speed grinding for natural peanut butter production. J. Food Eng..

[31] Codex Stan 152-1985. Codex Standard for Wheat Flour. www.fao.org/fao-who-codexalimentarius/sh-proxy/en/?lnk=1&url=https%253A%252F%252Fworkspace.fao.org%252Fsites%252Fcodex%252FStandards%252FCXS%2B152-1985%252FCXS_152e.pdf.

[32] Agimelen O.S., Mulholland A.J., Sefcik J. (2017). Modelling of artefacts in estimations of particle size of needle-like particles from laser diffraction measurements. Chem. Eng. Sci..

[33] Nathier-Dufour N., Bougeard L., Devaus M.-F., Bertrand D., de Monredon F.L.D. (1993). Comparison of sieving and laser diffraction for the particle size measurements of raw materials used in foodstuff. Powder Technol..

[34] Beals M.J., Fugal J.P., Shaw R.A., Lu J., Spuler S.M., Stith J.L. (2015). Holographic measurements of inhomogeneous cloud mixing at the centimeter scale. Science.

[35] Graham G., Nimmo-Smith A. (2010). The application of holography to the analysis of size and settling velocity of suspended cohesive sediments. Limnol. Oceanogr. Methods.

[36] Kumar S.S., He Z., Hogan C.J., Fredericks S.A., Hong J. (2020). Evaluation of laser diffraction-based particle size measurements using digital inline holography. Meas. Sci. Technol..

[37] Zhang K., Wong J.W., Krynitsky A.J., Trucksess M.W. (2014). Determining mycotoxins in baby foods and animal feeds using stable isotope dilution and liquid chromatography tandem mass spectrometry. J. Agric. Food Chem..

[38] Whitaker T.B., Springer J., Defize P.R., de Koe W.J., Coker R. (1995). Evaluation of sampling plans used in the United States, United Kingdom, and The Netherlands to test raw shelled peanuts for aflatoxin. J. AOAC Int..

[39] Champeil A., Fourbet J.-F., Doré T. (2004). Effects of Grain Sampling Procedures on *Fusarium* Mycotoxin Assays in Wheat Grains. J. Agric. Food Chem..

[40] Casado M.R., Parsons D., Weightman R., Magan N., Origgi S. (2009). Geostatistical analysis of the spatial distribution of mycotoxin concentration in bulk cereals. Food Addit. Contam. Part A Chem. Anal. Control. Expo. Risk Assess..

[41] Castells M., Marín S., Sanchis V., Ramos A.J. (2008). Distribution of fumonisins and aflatoxins in corn fractions during industrial cornflake processing. Int. J. Food Microbiol..

[42] Zhang K., Schaab M.R., Southwood G., Tor E.R., Aston L.S., Song W., Eitzer B., Majumdar S., Lapainis T., Mai H. (2017). A Collaborative Study: Determination of Mycotoxins in Corn, Peanut Butter, and Wheat Flour Using Stable Isotope Dilution Assay (SIDA) and Liquid Chromatography–Tandem Mass Spectrometry (LC-MS/MS). J. Agric. Food Chem..

[43] Zhang K. FDA Foods Program Compendium of Analytical Laboratory Methods: Chemical Analytical Manual (CAM). Method C-003.01. Determination of Mycotoxins in Corn, Peanut Butter, and Wheat Flour Using Stable Isotope Dilution Assay (SIDA) and Liquid Chromatography-Tandem Mass Spectrometry (LC-MS/MS). https://www.fda.gov/media/114240/download.

[44] Zhang K. (2020). Evaluation of Automated Sample Preparation for Mycotoxin Analysis in Foods. J. AOAC Int..

